# Preoperative Lung Ultrasound to Detect Pleural Adhesions: A Systematic Review and Meta-Analysis

**DOI:** 10.7759/cureus.14866

**Published:** 2021-05-06

**Authors:** Akihiro Shiroshita, Kiyoshi Nakashima, Masafumi Takeshita, Yuki Kataoka

**Affiliations:** 1 Department of Respiratory Medicine, Ichinomiyanishi Hospital, Ichinomiya, JPN; 2 Hospital Care Research Unit, Hyogo Prefectural Amagasaki General Medical Center, Amagasaki, JPN

**Keywords:** ultrasonography, systematic review, thoracic surgery, thoracoscopy

## Abstract

The usage of lung ultrasound as a preoperative examination for thoracic surgeries remains controversial. Our systematic review and meta-analysis aimed to evaluate preoperative lung ultrasound diagnostic accuracy for detecting pleural adhesions.

We searched articles published in MEDLINE, Embase, CENTRAL, and the International Clinical Trials Registry Platform until October 2020. Inclusion criteria were observational studies, case-control studies, and case series assessing preoperative lung ultrasound diagnostic accuracy. The study quality of included articles was evaluated using the modified quality assessment of diagnostic accuracy studies-2 tool. The pooled sensitivity and specificity were calculated using the bivariate random-effects model. The overall quality of evidence was summarized using the grading of recommendations, assessment, development, and evaluation approach.

Eleven articles were included in our systematic review. A high risk of bias was noted regarding undefined pleural adhesions and non-predefined pathological diagnosis. Based on the ten articles included for meta-analysis, the pooled sensitivity and specificity were 71% [95% confidence interval (CI), 56%-82%], and 96% (95% CI, 89%-99%), respectively. The overall quality of evidence was moderate.

Our systematic review revealed that lung ultrasound had high specificity. It may serve as a rule-in test for detecting pleural adhesions before thoracic surgeries, which may assist surgeons in preparation for a prolonged surgery or increased risk of complications that occurred by trocar insertion such as bleeding and persistent air leak.

## Introduction and background

Pleural adhesions between the visceral and pleural walls are a common condition encountered during thoracic surgeries, occurring in approximately 26% of the patients undergoing these procedures [1]. They are associated with increased mortality and operative time in thoracic surgery, especially during thoracoscopy [2]. Preoperative assessment of pleural adhesions may be important for the evaluation of surgical risk; further, based on the extent of pleural adhesions, surgeons may choose to modify the location of the trocar placement or convert a thoracoscopy procedure to an open thoracotomy [3].

To date, no gold standard technique has been established for identifying pleural adhesions before a thoracic surgery. Computed tomography (CT) is a static imaging modality and has proven to be difficult for the assessment of pleural adhesions (sensitivity, 72%; specificity, 71%) [4].

In contrast, ultrasound is a radiation-free, inexpensive, and quick imaging modality that can yield pleural excursion assessments in only five to 10 minutes [5]. An ultrasound can evaluate the pleura dynamically to visualize the absence of a lung sliding or gliding sign on B-mode images or the presence of a seashore or barcode sign on M-mode images. These findings are expected to facilitate the assessment of pleural adhesions [6-16]. However, the practical value of lung ultrasounds remains controversial as previous articles have been based on single-center studies with small sample sizes and unstandardized protocols. In this systematic review, we aimed to evaluate the diagnostic accuracy of the lung sliding sign in a lung ultrasound as a rule-in test for detecting pleural adhesions before thoracic surgery.

## Review

Methods

This study was designed as a systematic review and pre-registered in the International Prospective Register of Systematic Review (registration number: CRD42020212207). The reporting of this study is based on the preferred reporting items for systematic review and meta-analysis (PRISMA) for diagnostic test accuracy [17].

We performed a comprehensive search in MEDLINE, Embase, the Cochrane Library, and the International Clinical Trials Registry Platform until October 3, 2020, using related terms for lung ultrasound and pleural adhesion) (Table 1).

**Table 1:** The search strategy in each database

MEDLINE via Ovid	Search terms
Ultrasound	Exp Ultrasonography/or [(chest or lung* or thora* or pulm*) adj4 (sonogra* or ultrasound* or ultrasonic* or ultrasonogra* or ultra-sound* or ultra-sonic* or ultra-sonogra*)].ab,ti. or (sliding).ab,ti. or (gliding).ab,ti. or (seashore).ab,ti. or (barcode).ab,ti. or (stratosphere).ab,ti.
Pleural adhesion	(Pleural adhesion?).ab,ti. or (Intrathoracic adhesion?).ab,ti. or (Lung adhesion?).ab,ti.
Embase via Embase.com	
Ultrasound	"ultrasound"/exp OR [(chest OR lung* OR thora* OR pulm*) NEAR/4 (sonogra* OR ultrasound* OR ultrasonic* OR ultrasonogra* OR ultra-sound* OR ultra-sonic* OR ultra-sonogra*)]:ti,ab OR sliding:ab,ti OR gliding:ab,ti OR seashore:ab,ti OR barcode:ab,ti OR stratosphere:ab,ti
Pleural adhesion	"Pleural adhesion":ab,ti OR "Intrathoracic adhesion?":ab,ti OR "Lung adhesion?":ab,ti
Central	
Ultrasound	[Ultrasonography] explode all trees OR [(chest or lung* or thora* or pulm*) NEAR/4 (sonogra* or ultrasound* or ultrasonic* or ultrasonogra* or ultra-sound* or ultra-sonic* or ultra-sonogra*)]:ti,ab,kw OR (sliding):ti,ab,kw OR (gliding):ti,ab,kw OR (seashore):ti,ab,kw OR (barcode):ti,ab,kw OR (stratosphere):ti,ab,kw
Pleural adhesion	(Pleural adhesion?):ti,ab,kw OR (Intrathoracic adhesion?):ti,ab,kw OR (Lung adhesion?):ti,ab,kw
ICTRP	
	Adhesion AND Ultrasound

**Figure 1:**
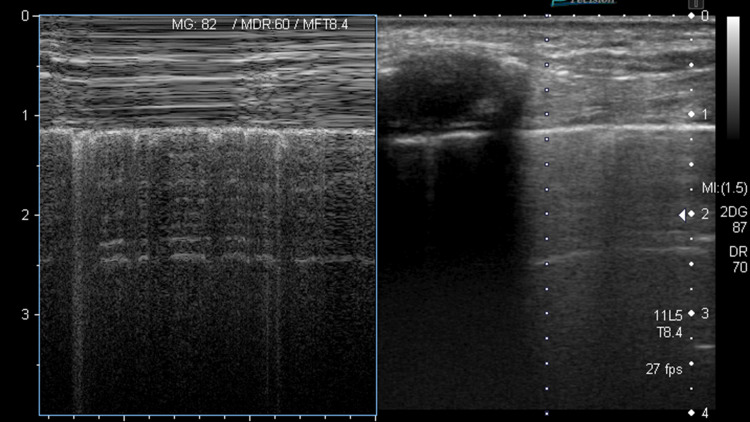
Seashore sign Seashore sign indicates normal lung sliding. "Sea" is derived from the straight lines created by the subcutaneous tissue and musculature. "Shore" is derived from sand-like appearance created by the continually moving aerated lung below the pleural line.

**Figure 2:**
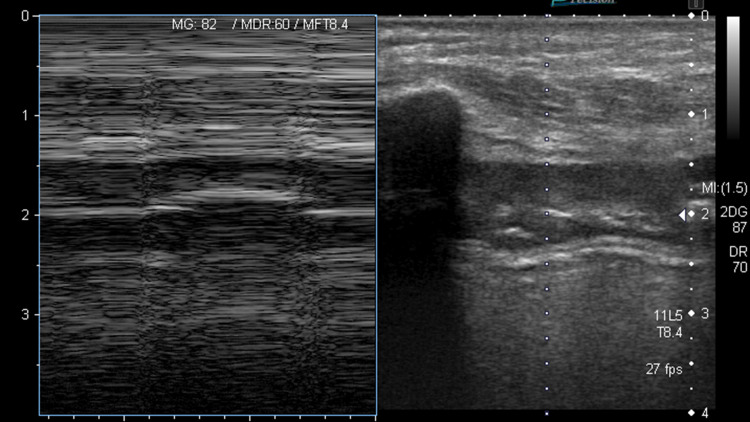
Barcode sign Barcode sign indicates absence of lung sliding ."Barcode" is derived from parallel horizontal lines below the pleural line.

Additionally, we searched for potentially relevant articles in the reference lists of the included articles and using a citation search in the web of science. Two reviewers (AS and KN) independently screened the eligible studies and then reviewed the full texts. We included prospective and retrospective observational studies, case-control studies, and case series with sufficient data to construct a two-by-two contingency table for the diagnostic yield of preoperative lung ultrasound in detecting pleural adhesions. There were no restrictions on language or publication status. Our target ultrasound findings were the absence of lung sliding or gliding sign on B-mode imaging, and absence of seashore sign, or presence of barcode or stratosphere sign on M-mode imaging (Figure 1 and Figure 2).

The sliding or gliding sign refers to the to-and-fro movement of the visceral pleura during breathing [18]; the absence of this finding indicates pleural adhesions. The seashore sign refers to a sand-like appearance beneath the pleural line, whereas the barcode or stratosphere sign refers to linear lines, both superficial and deep to the pleural line [19]. When pleural adhesions are present, the seashore sign is replaced by a barcode/stratosphere sign. As a reference standard, we accepted the confirmation of pleural adhesions by other modalities such as dynamic CT, or through macroscopic findings during thoracoscopy or thoracic surgery. We excluded articles describing emergent or urgent thoracic or cardiac surgery. Review articles, case reports, and animal studies were also excluded.

One of the two reviewers (AS and MT) extracted the data from the included articles, which was then double-checked. The information extracted from the articles was as follows: country, study design and setting, inclusion criteria, exclusion criteria, number of patients, patient's demographic characteristics, detailed methodology of lung ultrasound, information about the reference standard, and two-by-two contingency tables for diagnostic accuracy (true-positive, false-positive, false-negative, and true-negative). AS and KN evaluated the risk of bias and applicability using the Quality Assessment of Diagnostic Accuracy Studies (QUADAS-2) tool (Table 2) [20].

**Table 2:** Risk of bias and applicability assessment using the modified Quality Assessment of Diagnostic Accuracy Studies tool.

Domain	Patient selection	Index test	Reference standard	Flow and timing
Risk of bias				
Signaling questions	Was a consecutive or random sample of patients enrolled? Was a case-control design avoided? Did the study avoid inappropriate exclusions?^a^	Were the index test results interpreted without knowledge of the results of the reference standard? If a threshold was used, was it pre-specified?^b^	Is the reference standard likely to correctly classify the target condition?^c^ Were the reference standard results interpreted without knowledge of the results of the index test? Were the criteria of reference standard for target condition pre-specified?^d^	Was there an appropriate interval between index test(s) and reference standard?^e^ Did all patients receive a reference standard? Did patients receive the same reference standard? Were all patients included in the analysis?^f^
Explanations	a: Appropriate exclusions were defined as excluding patients who underwent emergency or urgent thoracic surgery, who had untreated pneumothorax, massive pleural effusion, diaphragmatic palsy, and patients who refused enrollment in the study. Inappropriate exclusions were defined as excluding patients based on antiplatelet use or based on the information of prior intrathoracic or breast surgeries, COPD, obesity, or gynecomastia.	b: The predefined definition of sliding sign was judged based on whether its cutoff points were predefined.	c: We accepted confirmation of pleural adhesions by other modalities than information during thoracic operation such as dynamic CT scan and macroscopic findings during medical thoracoscopy. d: The predefined pleural adhesions were judged whether the degree of pleural adhesions, such as dense and light, was predefined.	e: Appropriate interval between the ultrasound and operation was judged to be less than or equal to 60 days before operation. We decided “60 days” based on the recommendation for re-staging before surgery by the National Comprehensive Cancer Network (NCCN) Clinical Practice Guidelines in Oncology (NCCN Guidelines). f: We judged risk of bias based on whether missing data in the studies affected the diagnostic accuracy of ultrasound.
Applicability				
Signaling questions	Is there concern that the included patients do not match the review question?	Is there concern that the index test, its conduct, or interpretation differ from the review question?	Is there concern that the target condition as defined by the reference standard does not match the review question?	Not applicable

If disagreements arose between two reviewers during the review process, these were resolved by either a consensus or a consultation with a third reviewer (YK).

AS performed the statistical analyses using StataCorp. 2017. Stata Statistical Software: Release 15. College Station, TX: StataCorp LLC. and Review Manager (RevMan) [Computer program]. Version 5.4, The Cochrane Collaboration, 2020, and KY confirmed the process of the statistical analyses. First, we constructed a forest plot to illustrate the sensitivity and specificity in each study and evaluated the heterogeneity of diagnostic accuracy by calculating the I2 values. Then, regardless of the heterogeneity, we calculated the pooled sensitivity and specificity using the bivariate random-effects model. Additionally, the hierarchical summary receiver operating characteristic (HSROC) curve was generated. As an ad-hoc subgroup analysis, we calculated the sensitivity and specificity among patients with chronic obstructive pulmonary disease (COPD) and those with a body mass index (BMI) ≥ 30. As a sensitivity analysis, we calculated the sensitivity and specificity among studies that used only the B-mode images and in those with a high risk of bias. The overall quality of evidence for lung ultrasound was evaluated using the grading of recommendations, assessment, development, and evaluation (GRADE) approach [21].

Results

**Figure 3:**
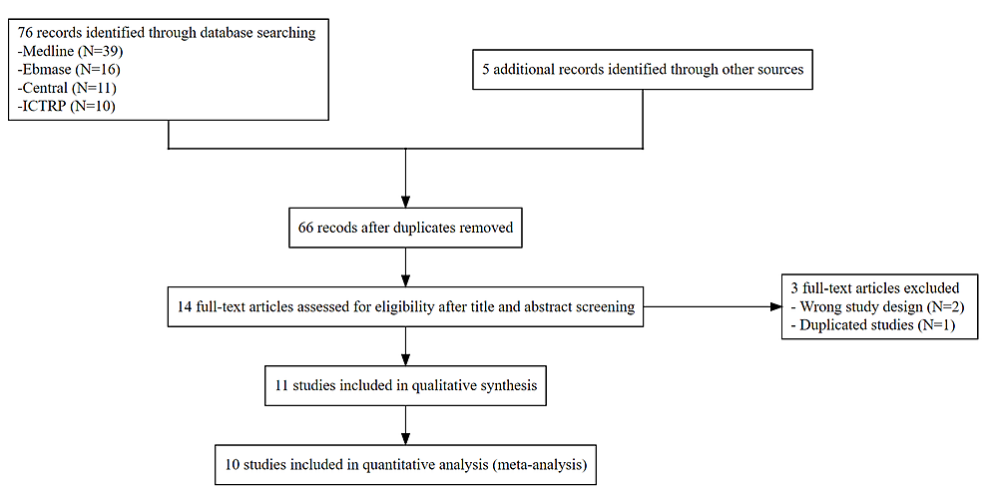
PRISMA flow chart The excluded articles after full-text screening were as follows: Wrong study design: Thiam K, Guinde J, Laroumagne S, Bourinet V, Berbis J, et al. Lateral decubitus chest radiography or chest ultrasound to predict pleural adhesions before medical thoracoscopy: a prospective study. J Thorac Dis. 2019 Oct;11(10):4292-4297. Nakano N, Oyama S, Kotake Y, Yasumitsu T. Ultrasonographic diagnosis of pleural adhesion in patients with lung cancer. JPN J Med Ultrason 1995;22:27-30. Duplicated studies: Jeong H, Ahn HJ, Lee EI, Gook JH. Usefulness of chest ultrasonography for prediction of pleural adhesion and postoperative severe pain in thoracoscopic surgery. J Cardiothorac Vasc Anesth. 2019 Sep;33:S114. (This is the conference abstract of Jeong 2020.)

Figure 3 shows the study selection process.

After screening 66 articles, we included 11 studies in the systematic review [6-16]. Information for the study by Shibasaki was obtained from the principal investigator via email [11]. We extracted the data from a total of 2366 sites in 840 patients who underwent elective thoracic surgery. Among these, 280 patients (33%) were women. All included studies had been performed at single centers. We have summarized the study characteristics in Table 3 and the lung ultrasound methodologies in Table 4.

**Table 3:** Study characteristics Abbreviations: SD, standard deviation; IQR, interquartile range; NA, not available

Study	Country	Study design	Procedures	Exclusions	Number of participants	Mean age (SD) or median [95% IQR]
Nakano, 1995 [6]	Japan	Prospective cohort study	Elective thoracotomy for lung tumor	None	125	64.1 (NA)
Tateishi, 2001 [7]	Japan	Retrospective cohort study	Elective thoracoscopy (volume reduction surgery for severe pulmonary emphysema, 11 patients; thoracoscopic esophagectomy for esophageal cancer, 6 patients; lobectomies for bronchogenic carcinoma, 4 patients; and extirpation of benign mediastinal neoplasms, 2 patients)	None	23	63 (NA)
Sasaki, 2005 [8]	Japan	Prospective cohort study	Scheduled thoracotomy or thoracoscopy	History of prior chest surgery	42	NA
Cassanelli, 2012 [9]	Italy	Prospective cohort study	Elective thoracotomy or thoracoscopy	Urgent surgery, untreated pneumothorax, massive pleural effusion, diaphragmatic palsy (78 cases excluded in total)	64	63 (13)
Wei, 2012 [10]	China	Prospective cohort study	Elective thoracotomy or thoracoscopy	None	117	59 (17)
Shibasaki, 2017 [11]	Japan	Prospective cohort study	Elective thoracoscopy, patient age ≥ 20 years	Pneumothorax	81	70 (12)
Eshraghi, 2019 [12]	Iran	Prospective cohort study	Thoracotomy and thoracoscopy surgery, patient age between 20 and 70 years	Heart disease such as dilated cardiomyopathy that affects the structure of the heart, BMI ≥ 35, or a history of gynecomastia or mastectomy in the past	96	45 (14)
Yasukawa 2019-1, [13]	Japan	Retrospective cohort study	Thoracoscopy for reoperations for ipsilateral pulmonary lesions	None	33	67 (14)
Yasukawa 2019-2, [14]	Japan	Retrospective cohort study	Thoracoscopy for aspirin users	Anticoagulation drugs and/or antiplatelet drugs other than aspirin	38	73 (7)
Homma, 2020 [15]	Japan	Prospective cohort study	Elective thoracic surgery	Pneumothorax, hydrothorax, hemothorax, pyothorax, chylothorax, age ≤ 19 or ≥90 years, median sternotomy, psychiatric disorders that inhibited participation, and inappropriate participation	168	69 [60–75]
Jeong, 2020 [16]	Korea	Prospective cohort study	Elective thoracoscopy. Age ≥ 19 years, and American Society of Anesthesiologists physical status Ⅰ to Ⅲ.	Pneumothorax, massive pleural effusion, emergency surgery	53	NA

**Table 4:** Detailed information for the lung ultrasound methodology Abbreviations: NA; not available, ICS; intercostal space

Study	Operator	Timing	Probe	Position	Probe site	Reference standard
Nakano, 1995 [6]	NA	NA	SSD 256 and 5-MHz linear probe	NA	Lateral sides	Macroscopic findings during surgical operation
Tateishi, 2001 [7]	NA	NA	LOGIC 500 or 700 scanner (GE Medical Systems; Milwaukee, WI) and a 7-MHz sector transducer	Sitting position	All ICSs	Operative videotapes and medical records
Sasaki, 2005 [8]	After initiation of the study, chest ultrasonography was interpreted by the consensus read of 1 thoracic surgeon and 1 radiologist for 3 patients. Afterwards, chest ultrasonography is performed by 2 thoracic surgeons for the remaining 39patients.	Within a week prior to the scheduled surgery	LOGIQ500 MR3plus (GE Yokogawa Medical Systems, Tokyo, Japan) 7-MHz array B-mode scanner.	Sitting position	Seven points in the ICSs. The midclavicular line in the 2nd ICS, the midaxillary and paravertebral lines in the 3rd ICS, the midaxillary and midclavicular lines in the 7th ICS, and the scapular lines in the 5th and 9th ICSs.	Macroscopic findings during surgical operation
Cassanelli, 2012 [9]	Two thoracic surgeons trained in TUS	On the day of the operation	Sonograph GE (Fairfield, CT, USA) Logiq® 7. A 3.5–5-MHz convex transducer in B- and M-modes	Sitting position	Seventeen lung segments	Macroscopic findings during surgical operation interpreted by a surgeon
Wei, 2012 [10]	One radiologist	Within 1 week prior to the scheduled surgery	a 7 L49 MHz linear transducer in B-mode	Lateral decubitus position lying on the non-affected side	6th ICS in the midaxillary line	Macroscopic findings during surgical operation interpreted by a surgeon
Shibasaki, 2017 [11]	Anesthesiologists	After tracheal intubation	Sonosite s nerve (Sonosite, Inc., Bothell, WA, USA), linear 13-6-MHz probe HFL38	Lateral position	3 or 4 Points marked by surgeons as port insertion sites	Macroscopic findings during surgical operation
Eshraghi, 2019 [12]	A single radiologist unit	NA	Ultrasonography device manufactured by Voluson, a US-based company, with a 7-10-5 MHz probe	NA	Seven points in the ICSs. The midclavicular line in the 2nd ICS, the midaxillary and paravertebral lines in the 3rd ICS, the midaxillary and midclavicular lines in the 7th ICS, and the scapular lines in the 5th and 9th ICSs.	Macroscopic findings during surgical operation interpreted by surgeons
Yasukawa 2019-1, [13]	NA	Within 2 weeks before the scheduled surgery	LOGIQ E9TM (GE Healthcare, Chicago, IL, USA) ultrasound system	Lateral position	The mid-axillary lines of the 7th or 8th ICSs	Macroscopic findings during surgical operation interpreted by surgeons
Yasukawa 2019-2, [14]	NA	Within 2 weeks before the scheduled surgery	LOGIQ E10TM (GE Healthcare, Chicago, IL, USA) ultrasound system	Lateral position	The mid-axillary lines of the 4th or 5th ICSs and the 7th or 8th ICSs	Macroscopic findings during surgical operation interpreted by surgeons
Homma, 2020 [15]	A chief surgeon	NA	A linear-type ultrasound probe (7.5 MHz) with a Prosound α7 scanner (Hitachi-Aloka medical, Ltd. Tokyo, Japan)	NA	Each ICS	Macroscopic findings during surgical operation
Jeong, 2020 [16]	2 anesthesiologists who had more than 3 years of experience in lung ultrasonography.	Before induction of anesthesia	Vivid S70N (GE Vingmed Ultrasound AS, Horten, Norway) with an 11 MHz linear transducer in B-mode and M-mode imaging	Supine position	The upper and lower blue points and the phrenic point, which are near the 2nd ICS in the midclavicular line, the 3rd ICS in the anterior axillary line, and lung-liver or lung-spleen junction at the midaxillary line	Macroscopic findings during surgical operation

Each article contained minimal information about the patients’ backgrounds. Data regarding the number of patients with COPD and obesity (BMI ≥ 30) could be obtained only from the studies by Cassanelli and Shibasaki [9, 11]. Lung ultrasound was primarily performed with two-dimensional B-mode imaging; M-mode imaging was only used in two studies as an additional modality when operators had difficulty viewing the pleura with B-mode imaging [9, 16]. In these two studies, we were unable to find the diagnostic accuracy of each ultrasound sign in either B-mode imaging or M-mode imaging. The macroscopic findings observed during surgery were used as a reference standard.

**Figure 4:**
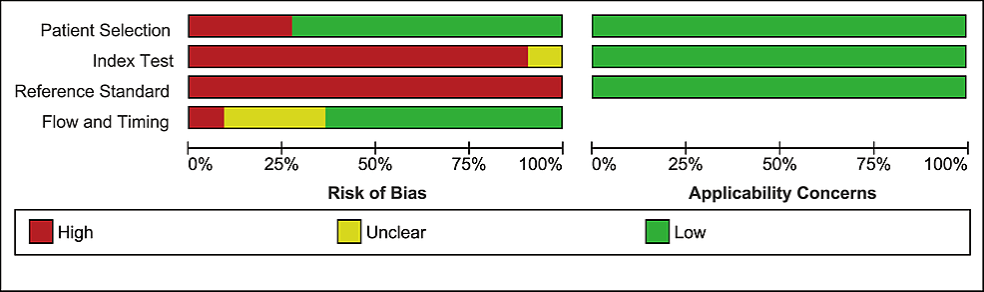
Methodological evaluation of thoracic ultrasound using the modified Quality Assessment of Diagnostic Accuracy Studies-2 tool The high risk-of-bias in the index test and reference standard domain was based on the unspecified methodology of the sliding sign, and not the pre-determined definition of pleural adhesions during thoracic operation. About half of the included articles were identified as having a high risk-of-bias in the patient selection domain due to inappropriate exclusion criteria.

**Figure 5:**
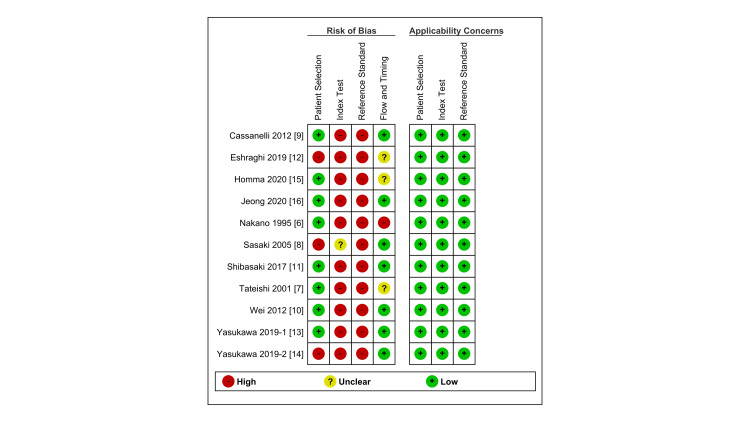
Assessment of risk of bias and applicability for each domain in the included studies Motoaki Yasukawa wrote 2 articles in 2019, “Yasukawa 2019-1” indicates “Yasukawa M, Taiji R, Marugami N, Kawaguchi T, Kawai N, Sawabata N, et al. Preoperative detection of pleural adhesions using ultrasonography for ipsilateral secondary thoracic surgery patients. Anticancer Res. 2019;39(8):4249-4252.” and “Yasukawa 2019-2” indicates “Yasukawa M, Taiji R, Marugami N, Kawaguchi T, Kawai N, Sawabata N, et al. Ultrasonography for detecting adhesions: Aspirin continuation for lung resection patients. In Vivo. 2019;33(3):973-8.”.

 We evaluated the quality of each study using the QUADAS-2 tool (Figure 4 and Figure 5).

A high risk of bias was identified in the domains of the index test and reference standard in all articles. Further, although Sasaki's study defined the cutoff point for a lung sliding sign, the article did not clarify whether the cutoff point was pre-defined [8]. In other articles, the cutoff points were not defined at all. The pleural adhesions were detected during thoracic surgery; however, a definition of pleural adhesion was provided only in the studies reported by Shibasaki and Yasukawa [11,14].

**Figure 6:**
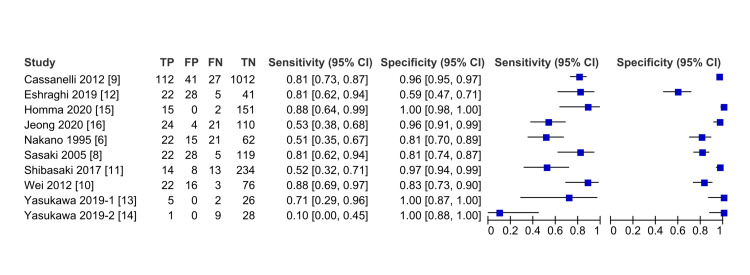
The forest plot of sensitivity and specificity in each study The forest plot revealed substantial heterogeneity among the included studies. Motoaki Yasukawa wrote 2 articles in 2019, “Yasukawa 2019-1” indicates “Yasukawa M, Taiji R, Marugami N, Kawaguchi T, Kawai N, Sawabata N, et al. Preoperative detection of pleural adhesions using ultrasonography for ipsilateral secondary thoracic surgery patients. Anticancer Res. 2019;39(8):4249-4252.” and “Yasukawa 2019-2” indicates “Yasukawa M, Taiji R, Marugami N, Kawaguchi T, Kawai N, Sawabata N, et al. Ultrasonography for detecting adhesions: Aspirin continuation for lung resection patients. In Vivo. 2019;33(3):973-8.”

We constructed a forest plot of sensitivity and specificity for each study, and the heterogeneity of the studies was substantial (I2 = 97, 95% confidence interval [CI], 94-99) (Figure 6).

**Figure 7:**
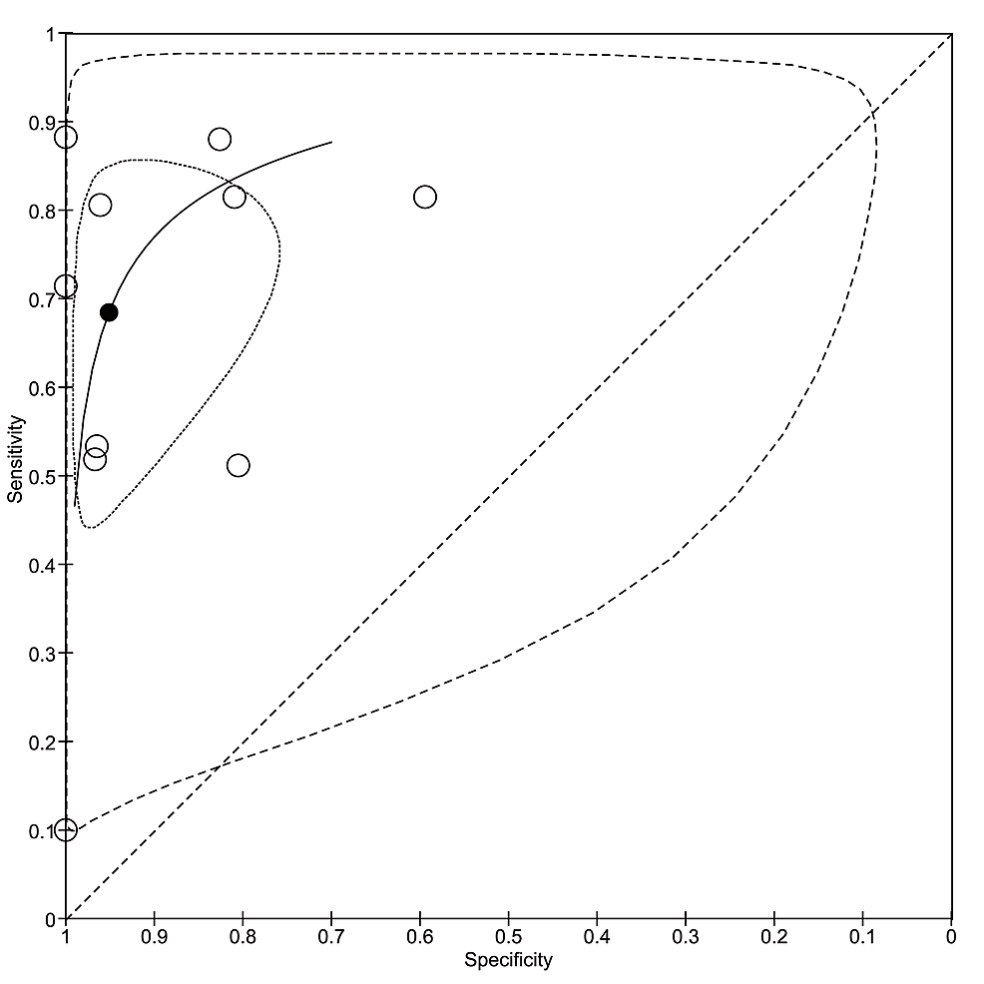
The hierarchical summary of the receiver operating characteristics curve of lung ultrasound The black circle shows the bivariate summary estimates, the inner dotted line indicates the 95% confidence region, and the outer dotted line indicates the 95% prediction region. The hierarchical summary receiver operating characteristic curve is illustrated taking into account of within- and between-study heterogeneity with correlation between sensitivity and specificity. The 95% confidence region shows an uncertainty of the summary sensitivity and specificity while the 95% prediction region shows potential values of sensitivity and specificity that could be observed in a future study.

We included 10 out of 11 studies in the meta-analysis as the sensitivity and specificity of sliding signs alone were not described in the study by Tateishi [22]. The pooled sensitivity and specificity of lung ultrasound was 68% (95% CI, 53%-81%) and 95% (95% CI, 85%-98%), respectively. Figure 7 illustrates the HSROC curve of lung ultrasound for detecting pleural adhesions.

We did not perform an a priori subgroup analysis among patients with COPD or obesity due to a scarcity of information. Our sensitivity analysis targeting studies with a low risk-of-bias in the patient-selection domain and those that underwent only B-mode imaging showed moderate sensitivity and high specificity (pooled sensitivity and specificity, 71% [95% CI, 56%-82%] and 96% [95% CI, 89%-99%], respectively, in low-risk-bias studies; and 68% [95% CI, 48%-83%] and 95% [95% CI, 79%-99%], respectively, in B-mode imaging only studies). The overall quality of evidence of lung ultrasound for detecting pleural adhesions, evaluated using the GRADE approach, revealed a moderate certainty of evidence (Table 5).

**Table 5:** Findings with pleural adhesions via lung ultrasound using the Grading of Recommendation, Assessment, Development and Evaluation approach a. In most of the included studies, the definitions of the lung sliding sign and pleural adhesions as reference standards were not provided. Approximately half of the included studies excluded patients with prior thoracic surgeries, those taking anticoagulation or antiplatelet drugs, and patients with obesity, gynecomastia, or a prior mastectomy.

Should lung ultrasound be used to detect pleural adhesion before thoracic surgeries? Patients: Patients who plan to undergo elective thoracic surgeries Setting: Before elective thoracoscopy or thoracotomy Index test: Lung ultrasound including the B-mode images (absence of sliding sign or gliding sign) and the M-mode images (absence of seashore sign, or presence of barcode sign or stratosphere sign) Reference test: The confirmation of pleural adhesions by other modalities such as dynamic computed tomography scan, or macroscopic findings during surgical operation. Pooled sensitivity: 68% (95% CI, 53%–81%) | pooled specificity: 95% (95% CI, 85%–98%)											
Outcome	Number of studies (number of patients)	Study design	Factors that may decrease the certainty of evidence					Effect per 1,000 patients tested			Test accuracy certainty of evidence
			Risk of bias	Indirectness	Inconsistency	Imprecision	Publication bias	Pre-test probability of 50%	Pre-test probability of 30%	Pre-test probability of 10%	
True positives (patients with pleural adhesions)	11 studies 840 patients	Cohort & case-control type studies	Serious ^a^	Not serious	Not serious	Not serious	None	340 (265-405)	204 (159-243)	68 (53-81)	⨁⨁⨁◯ MODERATE
False negatives (patients incorrectly classified as not having pleural adhesions)								160 (95-235)	96 (57-141)	32 (19-47)	
True negatives (patients without pleural adhesions)	11 studies 840 patients	Cohort & case-control type studies	Serious ^a^	Not serious	Not serious	Not serious	None	475 (425-490)	665 (595-686)	855 (765-882)	⨁⨁⨁◯ MODERATE
False positives (patients incorrectly classified as having pleural adhesions)								25 (10-75)	35 (14-105)	45 (18-135)	

Discussion

**Figure 8:**
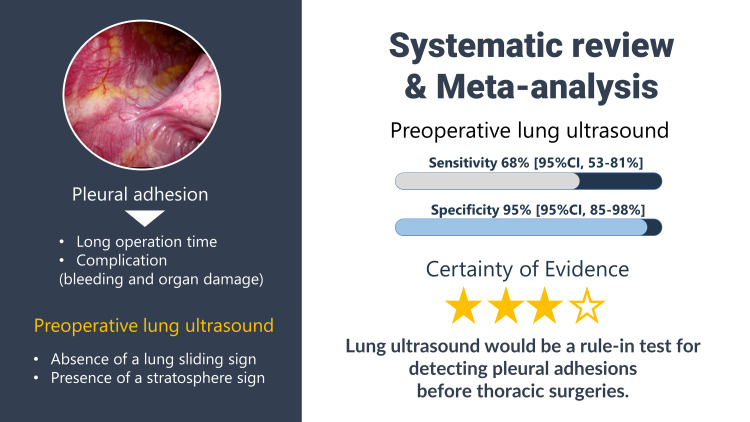
Graphical Abstract

We aimed to evaluate whether lung ultrasound may be performed as a rule-in test for detecting pleural adhesions before thoracic surgeries. Our systematic review and meta-analysis showed that lung ultrasound had a high specificity for detecting pleural adhesions before thoracic surgery. Further, the sensitivity of lung ultrasound was observed to be similar to that of CT [4]. Based on the moderate overall quality of evidence, further prospective studies with a definition for pleural adhesions and pre-defined lung sliding signs are needed. Our systematic review and meta-analysis showed that lung ultrasound had a high specificity for detecting pleural adhesions before thoracic surgery [Video 1 and Figure 8]

**Video 1:** Video Abstract This video abstract was produced by Akihiro Shiroshtia. Our systematic review revealed that lung ultrasound may serve as a rule-in test for pleural adhesions before thoracic surgeries. Based on the results of the ultrasound, surgeons may be able to prepare for a prolonged duration of surgery and a high risk of associated complications.

The high specificity of lung ultrasound supported the diagnosis of pleural adhesions with certainty, and the absence of a sliding sign indicated dense rather than loose pleural adhesions [15]. This information may be used to identify high-risk patients in order to prepare the patient and medical team for prolonged surgeries and the associated complications [2]. In cases where pleural adhesions are identified, changing the trocar insertion site or converting to open thoracotomy may be considered to reduce the risk of bleeding and persistent air leak.

The sensitivity of lung ultrasound was observed to be insufficient to rule out pleural adhesions. However, this may be attributable to the experience level of the operators and the criteria used to identify pleural adhesions. Except for the studies by Cassanelli and Jeong, the remaining studies included in this systematic review did not provide information about the operators’ level of experience [9,16]. Ultrasound is a relatively new and operator-dependent imaging modality in the field of thoracic surgery [22], and it requires operators with extensive knowledge, experience, and a high level of skill. Since lung sliding signs have been used widely in patients with pneumothorax (sensitivity, 88%; specificity, 99%), operators may need to improve their sensitivity in detecting pleural adhesions in such cases [23]. Notably, pleural adhesions were not defined in nearly all of the included articles. Distinguishing between and defining dense vs light (“spider web”) adhesions on ultrasound with good sensitivity and specificity is of clinical importance, as 24% of severe or dense adhesions resulted in postoperative bleeding, compared with only 5% of light or “spider web” adhesions. Therefore, the focus in future studies should be on dense adhesions.

Among the included articles, the cutoff visceral slide distance was described only in the study by Sasaki [8]. Preoperative assessment of peritoneal adhesions is usually based on whether the distance is less than 2 cm [24,25]. In the upper thoracic cage, pleural movement can be difficult to assess because of the greater distance in the upper thoracic cage between the probe and the pleura than that in the lower thoracic cage [8,10]. Thus, a cutoff of 1 cm in the upper thoracic cage and 2 cm in the lower thoracic cage may be reasonable, as described by Sasaki [8]. In addition, the probe sites were different between studies and not strictly defined. To date, there are some standardized protocols in other fields. In the field of emergency medicine, a standardized protocol for immediate diagnosis of acute respiratory failure was proposed by Lichtenstein et al [26]. It is a simple protocol composed of three sites (upper, lower, and posterolateral alveolar and/or pleural syndrome point) in each lung based on the size of the patient’s two hands [27]. In addition, a computerized program has been evolving for detecting pneumothorax automatically [28]. For a standardized protocol, we may need further studies assessing the diagnostic yield of different cutoff points with standardized probe sites.

This systematic review has several limitations. First, the studies contained minimal background information regarding the patients. Moreover, although lung ultrasound showed high specificity, we were not able to calculate the sensitivity and specificity in specific, more complicated populations, such as those with COPD or obesity. COPD is a major cause of diminishing lung sliding signs due to overinflation of the lungs [26], and the resolution of ultrasound is reduced in obese patients due to excessive fatty tissue [22]. However, among the reviewed studies, 510 out of the 840 patients (61%) were Japanese individuals with a relatively low BMI [29]. Although we could not estimate why most of the included studies were from Asian countries, a selection from a particular region could limit the generalizability of this review. Second, we were unable to directly assess the patient-reported outcomes. Further studies with larger sample size, especially including patients with COPD and obesity from non-Asian countries, are needed to evaluate patient-reported outcomes and surgical complications, including surgery duration and bleeding.

## Conclusions

In conclusion, lung ultrasound may serve as a rule-in test for pleural adhesions before thoracic surgery, allowing surgeons to prepare in advance for a prolonged surgery and a high risk of complications, and avoiding certain complications such as bleeding and persistent air leak that can occur as a result of trocar insertion through pleural adhesions.
